# Implementing the Digital Diabetes Questionnaire as a Clinical Tool in Routine Diabetes Care: Focus Group Discussions With Patients and Health Care Professionals

**DOI:** 10.2196/34561

**Published:** 2022-05-25

**Authors:** Maria Svedbo Engström, Unn-Britt Johansson, Janeth Leksell, Ebba Linder, Katarina Eeg-Olofsson

**Affiliations:** 1 School of Health and Welfare Dalarna University Falun Sweden; 2 Center for Clinical Research Dalarna Uppsala University Falun Sweden; 3 Sophiahemmet University Stockholm Sweden; 4 Department of Clinical Sciences and Education Södersjukhuset Karolinska Institutet Stockholm Sweden; 5 Department of Medical Sciences Clinical Diabetology and Metabolism Uppsala University Uppsala Sweden; 6 Swedish National Diabetes Register Center of Registers Västra Götaland Gothenburg Sweden; 7 Sahlgrenska Academy Gothenburg University Gothenburg Sweden; 8 Sahlgrenska University Hospital Gothenburg Sweden

**Keywords:** diabetes mellitus, type 1, diabetes mellitus, type 2, focus groups, health care professionals, outpatients, patient care, patient participation, patient-reported outcome measures, qualitative research, registries

## Abstract

**Background:**

The Diabetes Questionnaire is a digital patient-reported outcome and experience measure for adults living with diabetes. The Diabetes Questionnaire is intended for use in routine clinical visits in diabetes care and to enable patient perspectives to be integrated into the Swedish National Diabetes Register. The Diabetes Questionnaire was developed on the basis of patients’ perspectives, and evidence for its measurement qualities has been demonstrated. Patients receive an invitation to complete the questionnaire before clinical visits, and the patient and health care professional (HCP) can discuss the findings, which are instantly displayed during the visit. Implementation processes for new tools in routine care need to be studied to understand the influence of contextual factors, the support needed, and how patients and HCPs experience clinical use.

**Objective:**

The aim of this study was to describe patients’ and HCPs’ experiences of initiating the use of the digital Diabetes Questionnaire as a clinical tool in routine diabetes care, supported by a structured implementation strategy involving initial education, local facilitators, and regular follow-ups.

**Methods:**

In this qualitative study, semistructured focus group discussions were conducted 12 months after the use of the Diabetes Questionnaire was initiated. Participants were diabetes specialist nurses and physicians (20 participants in 4 groups) at hospital-based outpatient clinics or primary health care clinics and adults with type 1 or type 2 diabetes (15 participants in 4 groups). The audiotaped transcripts were analyzed using inductive qualitative content analysis.

**Results:**

The results revealed 2 main categories that integrated patients’ and HCPs’ experiences, which together formed an overarching theme: *While implementation demands new approaches, the Diabetes Questionnaire provides a broader perspective*. The first main category (*The Diabetes Questionnaire supports person-centered clinical visits*) comprised comments expressing that the digital Diabetes Questionnaire can initiate and encourage reflection in preparation for clinical visits, bring important topics to light during clinical visits, and broaden the scope of discussion by providing additional information. The second main category (*The process of initiating the implementation of the Diabetes Questionnaire*) comprised comments that described differences in engagement among HCPs and their managers, challenges of establishing new routines, experiences of support during implementation, thoughts about the Diabetes Questionnaire, need to change local administrative routines, and opportunities and concerns for continued use.

**Conclusions:**

The Diabetes Questionnaire can broaden the scope of health data in routine diabetes care. While implementation demands new approaches, patients and HCPs saw potential positive impacts of using the questionnaire at both the individual and group levels. Our results can inform further development of implementation strategies to support the clinical use of the questionnaire.

## Introduction

### Background

The Diabetes Questionnaire is a digital patient-reported outcome and experience measure (patient-reported outcome measure [PROM] and patient-reported experience measure [PREM]) for adults living with diabetes. This measure is primarily designed for use in clinical visits, but can also be used to enable patient perspectives to be integrated into the Swedish National Diabetes Register (NDR). The questionnaire was developed on the basis of patients’ perspectives, and evidence for its measurement qualities has been demonstrated. Stemming from 33 items, the questionnaire generates scores from 0 to 100 on dimensions such as general well-being, mood and energy, freedom from worries, management of daily life activities, and experiences of support from diabetes care [1-5]. Patients receive an invitation to complete the questionnaire before clinical visits, and the patient and health care professional (HCP) can discuss the findings, which are instantly displayed during the visit. Thus, the Diabetes Questionnaire has the potential to facilitate patient participation and support steps toward person-centered care [2].

Patient participation and person-centered care are emphasized in the guidelines for diabetes care [6-15] and Swedish legislation [16]. In addition, the inclusion of patients’ perspectives in the outcomes of clinical diabetes care has been encouraged in recent decades [6-9,12,17-19]. Although results from randomized controlled trials are limited, it has been suggested that user-friendly PROMs used in routine practice can strengthen the patient’s role, centralize information [20-23], and facilitate improvements in diabetes care [20]. Compared with paper-and-pen questionnaires, digital tools have been found to be quicker and easier to use for administration, completion, and presentation of results and have lower costs and better data quality [24]. Furthermore, the possibility of visualizing results during clinical visits has been found to facilitate insight into the patient’s situation and improve communication between patients and HCPs [24]. Additional research is needed to learn more about the perspectives of HCPs and patients regarding the implementation and use of PROMs in clinical practice [25-27].

The implementation of a new tool such as the digital Diabetes Questionnaire in a clinical setting is challenging and needs to be undertaken with caution and in a structured manner. Implementation processes need to be studied to learn about contextual influencing factors, required support, and how patients and HCPs experience clinical use [28]. To study the initial implementation process, we conducted a 2-part qualitative study. The first part of the study [29] addressed patients’ and HCPs’ perceptions and attitudes about implementing the digital Diabetes Questionnaire in routine diabetes care before the implementation was started. The findings indicated the potential usefulness of the Diabetes Questionnaire to support a more person-centered approach to care and for patients to reflect on their situation and everyday life with diabetes. Expressing hopes and concerns about digital technology in general, the participants emphasized the need for HCPs to be trained in practically handling the digital Diabetes Questionnaire and addressing patients’ questionnaire responses [29]. This paper describes the second part of the study, focusing on experiences after initiating the implementation of the digital Diabetes Questionnaire as a clinical tool for routine diabetes care using a structured implementation strategy. Inspired by Moore et al [28], this implementation strategy included introductory information for patients, education for HCPs about the digital Diabetes Questionnaire and its administrative tools, and engagement of local facilitators to support the work with regular follow-up.

### Aim of the Study

The aim of this study was to describe patients’ and HCPs’ experiences of initiating the use of the digital Diabetes Questionnaire as a clinical tool in routine diabetes care, supported by a structured implementation strategy involving initial education, local facilitators, and regular follow-up.

## Methods

### Research Design

A descriptive qualitative design was used in this follow-up study. The interviews were conducted through focus group discussions with HCPs and adult patients with diabetes.

### Participants and Setting

Participants were recruited through purposive sampling from the same sample that was included in the first part of this qualitative study [29]. Of the initial 14 hospital-based outpatient clinics and 8 primary health care clinics that participated in the first part of the study (52 individuals), 13 (93%) hospital clinics and 6 (75%) primary health care clinics had the possibility to participate in the second part of the study. The clinics were active users of the NDR and were located in different regions in Sweden. Diabetes specialist nurses, physicians, and adult patients with type 1 or type 2 diabetes were included in a total sample of 35 participants.

### The Implementation Strategy

Inspired by Moore et al [28], the structured implementation strategy for initiating the use of the digital Diabetes Questionnaire as a clinical tool in routine diabetes care included initial education for HCPs and patients about the questionnaire and the digital tool for administering and answering the questionnaire. The strategy also included the engagement and education of local facilitators to support clinics with regular follow-up. The implementation strategy is outlined in Textbox 1.

**The implementation strategy.**

**Initial education in respective groups**

- Health care professionals (HCP)
  Short film introduction on the Diabetes Questionnaire—including patients’ and HCPs’ perspectives
  Information about the concepts of patient-reported outcome and experience measures (patient-reported outcome measures and patient-reported experience measures)
  Background and overview of how the Diabetes Questionnaire was developed and constructed with questions and dimensions
  Preparatory training on how to send and receive the answered questionnaires using the digital tool for administering the questionnaire
  Examples of how questionnaire responses are presented and can be discussed at clinical visits
- Patients
  Short film introduction on the Diabetes Questionnaire—including patients’ and HCPs’ perspectives
  Information about the concepts of patient-reported outcome measure and patient-reported experience measures
  Background and overview of how the Diabetes Questionnaire was developed and constructed with questions and dimensions
  Preparatory training on how to log in and answer the digital questionnaire
  Examples of how the responses are presented

**Education for local facilitators**

- Initial education as in the HCP group
- Information on how to facilitate, for example, to contact the local clinics, offer support, and be available to answer questions from the clinics

**Regular follow-ups**

- Conducted by facilitators
  The facilitators contacted all the participating clinics by email at least once during the study to support and provide the HCPs the opportunity to discuss various problems or thoughts regarding the use of the questionnaire
  The facilitators offered additional contact and support according to the clinic’s needs
- Conducted by study team
  Follow-up contact with the local facilitators during the study
  Follow-up to the local facilitator regarding the clinics’ sent and received questionnaires

### Data Collection

A total of 8 focus groups were conducted. Of these, 50% (4/8) were conducted with adult patients living with either type 1 or type 2 diabetes and 50% (4/8) were conducted with HCPs. Background data are presented in Table 1. In accordance with the implementation strategy and study follow-up time of 12 months, both patients and HCPs had acquired initial experience in using the Diabetes Questionnaire. At the time of the focus group discussions, all HCPs had sent invitations to patients to answer the questionnaire and had discussed the answers with patients more than once. Most of the participating HCPs had undertaken several such conversations with different patients, but the number of conversations differed between the HCPs. As anticipated, the participating patients answered only 1 questionnaire since being invited to participate in this study, because routine clinical visits occur only once or a few times in a year. All patients except 1 (14/15, 93%) were given the opportunity to answer the Diabetes Questionnaire on 1 occasion. All patients who answered the questionnaire followed up the answers with their nurse or physician, some during clinical visits and some by telephone.

**Table 1:** Background data of the focus groups.

Focus group and characteristics		Patients with diabetes^a^ (n=15)	Health care professionals^b^ (n=20)
Groups, n		4	4
**Sex, n (%)**			
	Male	8 (53)	5 (25)
	Female	7 (47)	15 (75)
**Diabetes type, n (%)**			N/A^c^
	Type 1	11 (73)	
	Type 2	4 (27)	
**Profession, n (%)**		N/A	
	Diabetes specialist nurse		16 (80)
	Physician		4 (20)

^a^All except 1 patient (14/15, 93%) had answered 1 questionnaire before the focus group discussion.

^b^In total, 1465 questionnaire invitations were sent and 577 (39.39%) answered questionnaires were received before the focus group discussion.

^c^N/A: not applicable.

The focus group discussions were based on semistructured interview guides for the patient and HCP groups (Multimedia Appendix 1). Follow-up questions such as “Could you please further describe the situation using a concrete example?” were used as needed. KEO and JL moderated the focus groups, and EL facilitated the discussions. The focus group discussions were conducted at hospital-based outpatient clinics, primary health care clinics, and the Center of Registers Västra Götaland. The discussions lasted between 0.8 and 1.2 hours, were audio recorded with a digital voice recorder, and subsequently transcribed by a medical secretary.

### Data Analysis

The interviews were analyzed using qualitative content analysis [30], with an inductive approach. Each interview was considered as the unit of analysis. The verbatim transcripts (221 pages; 78,955 words) were read several times to identify the essential features (EL, KEO, and MSE). EL, KEO, and MSE agreed that data saturation was satisfactory as the material was nuanced and rich and that there were repetitive contents between transcripts. Units of meaning were identified (EL and KEO), condensed, and labeled with descriptive codes similar to the wording in the text. The codes from all the interviews were assembled and grouped into subcategories according to their content. Continuing the abstraction process, subcategories were pooled into categories and main categories and given an overarching descriptive theme. Each category was based on codes that were judged to belong together and collectively form the basis of meaningful content that was different from that of other categories. Researcher triangulation was used to discuss each step of the analysis process, moving back and forth as needed (EL, KEO, and MSE), finishing with a discussion to reach consensus about the categorization and theme between all authors (EL, JL, KEO, MSE, and UBJ). The analysis process was conducted manually using a word-processing program (Word; version 2202; Microsoft 365). The use of figure labels enabled the back-and-forth process while keeping track of each text segment throughout the analysis.

### Ethics Approval

The Swedish Ethical Review Authority in Gothenburg (317-18) approved the study. A letter provided to participants informed them about the study’s purpose, voluntary nature of their participation, confidentiality measures and methods of handling their personal data, NDR, contact details, and right to withdraw consent at any time and with immediate effect without specifying a reason. This information was also provided verbally at the beginning of each focus group. All participants provided written informed consent, and the study was completed in accordance with the Declaration of Helsinki [31].

## Results

### Overview of Main Categories and Theme

The 2 main categories that emerged in the analysis were the following: *The Diabetes Questionnaire supports person-centered clinical visits* and *The process of initiating the implementation of the Diabetes Questionnaire*. These main categories constituted the overarching theme, *While implementation demands new approaches, the Diabetes Questionnaire provides a broader perspective* (Textbox 2). The main categories are described in the following sections, with exemplifying quotes.

**Theme, main categories, and categories.**

**Theme**

- While implementation demands new approaches, the Diabetes Questionnaire provides a broader perspective

**Main category 1**

- The Diabetes Questionnaire supports person-centered clinical visits

**Categories**

- Initiating preparation and reflection before clinical visits
- Bringing important topics to light during clinical visits
- Broadening understanding by providing new information

**Main category 2**

- The process of initiating the implementation of the Diabetes Questionnaire

**Categories**

- Differences in engagement among health care management and coworkers
- Starting and establishing new routines
- Health care professionals’ experiences of support during implementation of the questionnaire
- Pros and cons regarding the questionnaire and its items and dimensions
- Administration and completion of the Diabetes Questionnaire
- Future opportunities and concerns

### The Diabetes Questionnaire Supports Person-Centered Clinical Visits

#### Initiating Preparation and Reflection Before Clinical Visits

Participants described the Diabetes Questionnaire as a tool that initiated preparation and enabled reflection in preparation for clinical visits, for both HCPs and patients. Patients and HCPs expressed that the completion of the questionnaire and obtaining dimension scores encouraged patients to reflect on aspects of their everyday life and their care that were not working well and actively prepare topics to discuss during clinical visits. A patient expressed the following:

Completing the questionnaire made me take time to sit down and reflect about what it really was [that I wished to discuss]. This was different to the usual routine of just showing up at the clinic.

patient, group 4

Similarly, patients and HCPs discussed the ways in which HCPs could prepare for clinical dialogue by reflecting on their patients’ scores.

#### Bringing Important Topics to Light During Clinical Visits

Although some HCPs found that the Diabetes Questionnaire was just another way of identifying topics that were already addressed, patients and HCPs expressed that the Diabetes Questionnaire can be a valuable tool for bringing important topics to light in the dialogue during clinical visits. Patients and HCPs emphasized the need to discuss the questionnaire scores and for the patients to explain and describe the reasons for their ratings. HCPs understood from the dialogue that patients had been reflecting on their ratings, but also expressed that not all patients wanted to talk about their scores or problems and that this choice must be respected. Patients reported 2 prerequisites for a good discussion: that they had completed the questionnaire beforehand and that they had completed it recently enough that the responses were still relevant and they remembered the reasoning behind their responses. Patients found the Diabetes Questionnaire to be a useful tool for remembering and prioritizing topics they felt were important to discuss, because clinical visits are time-constrained. Patients expressed that they also wanted time to discuss topics other than those raised in the questionnaire:

The time for clinical visits is limited. The questionnaire is a helpful aid for getting to the questions you need to discuss.

patient, group 3

Patients discussed how the dialogue related to the Diabetes Questionnaire could broaden HCPs’ understanding of their patients as human beings. HCPs discussed how working with the questionnaire made them better at taking a step back, lecturing less, and encouraging patients to talk about the topics that are important to them. This helped HCPs better understand their individual patients’ perspectives, the problems their patients experience, and what life with diabetes can be like. HCPs also discussed how the questionnaire helped patients realize that it is appropriate to talk about their personal experiences and topics other than medical matters and that talking to an HCP can be particularly valuable for patients who do not have anyone in their social sphere who understands what they are going through. HCPs found that using the questionnaire meant that clinical visits were more likely to be on the patient’s terms by facilitating joint discussion and encouraging patients to be more active in speaking their minds, which enhanced patient participation and more individualized care. Patients discussed how the Diabetes Questionnaire facilitated dialogue with HCPs and increased the extent to which HCPs behaved in a supportive manner. However, patients found that the responsibility for everyday self-management was not affected and still rested with the individual with diabetes:

I’m the only person who can do something about it. My diabetes nurse and physician can’t solve all of my problems. I see them as tools for support for when something isn’t working and I don’t understand why. As for the rest of it, I’m in charge. That’s how it was both before and after the questionnaire, so it doesn’t affect that.

patient, group 1

Patients and HCPs stated the importance of discussing about questionnaire scores even if the problems were not directly related to diabetes. Patients and HCPs had similar experiences that general well-being and difficulties in everyday life impact individuals with diabetes and affect their ability to manage their condition. Sometimes, HCPs encountered patients with dimension scores that were higher or lower than anticipated, thus increasing the importance of dialogue and the need to obtain more information from their patients. This process was described as a balancing act with a need for dialogue when the medical parameters indicated problems but the questionnaire scores did not. Patients and HCPs expressed that the questionnaire was most needed when changes were made, for meetings with patients who were less communicative, or for meetings between patients and HCPs who had not met previously. HCPs suggested that the questionnaire may become more important given the increasing focus on technology and quantitative data, which they described as potentially receiving a lot of attention:

Perhaps it will become more and more important because the role of technology is increasingly dominant in clinical visits. The focus is very much on graphs [showing data from glucose sensors] and insulin pumps and so on. It’s easy to forget about the most important question: ‘How are you doing, really?’

HCP, group 1

#### Broadening Understanding by Providing New Information

Patients and HCPs discussed how the Diabetes Questionnaire could broaden the scope of understanding by providing more information, deepening the dialogue, and leading to new insights. Although the received information did not always contain new insights, participants found that the questionnaire provided a more nuanced picture of their individual situation by raising topics in clinical dialogue that may not have been addressed previously, despite several years of contact in many cases. HCPs expressed that this new focus helped them and their patients to see the situation from other points of view. In addition, the questionnaire revealed to HCPs and patients the different aspects of an individual’s life that have an impact on the patient’s self-managed treatment and medical outcomes. This perspective enabled HCPs to understand that the patient’s general well-being was of special importance. The questionnaire was helpful for focusing on the most important topics by pinpointing what patients themselves saw as their most pressing problems and needs and where there was scope for improvement. Both patients and HCPs remarked that the perspectives of patients and diabetes care staff may differ. Patients expressed that it can be difficult for them to convey their needs and problems if they have doubts about the HCP’s interest in those topics, but that the Diabetes Questionnaire affirmed the importance of the patients’ perspective, and the related dialogue helped them realize that HCPs wanted to help them:

When you have this questionnaire, there’s an opportunity. They’re saying ‘We want to help you, fill it in, be honest.’ [...] After all, perhaps you do want help with something...It definitely changed my perspective.

patient, group 4

Patients appreciated the questionnaire for taking their overall life situation into account and discussed it as a means for themselves and their HCPs to recognize and talk about how they may not feel as good as they would like to or as they pretend to. HCPs also discussed having learned that, despite its challenges, diabetes can be experienced as less of a barrier to a good life than they initially thought. HCPs discussed their experiences of having observed discrepancies between medical parameters and patient perspectives that were previously unknown to them, sometimes leading to new insights. For example, HCPs described having learned that patients with well-controlled blood glucose levels, who they thought were doing well, may have problems such as diminished general well-being, being hampered by diabetes in their everyday life, struggling with worries about diabetes-related everyday security, and future risks of long-term complications. The following was expressed by an HCP:

‘You’re the ideal patient with no problems.’ That’s what it can feel like when you look at a patient’s medical records. But the patient might actually be very limited by their illness in their everyday life.

HCP, group 4

Other issues that were experienced as being highlighted by the questionnaire were patients’ feelings of loneliness; experiences of lack of support from family, friends, and colleagues; and lack of employment security. Both patients and HCPs realized that HCPs are often unaware of these types of lack of support. Some patients expressed that they initially considered lack of social support to be their own responsibility to cope with, but that they were prompted to think about it more when they noticed that their HCP was concerned about it. By addressing these issues, patients suggested that the Diabetes Questionnaire could be a tool for involving their significant others in their diabetes care and to highlight the needs of significant others in terms of receiving support from diabetes care staff.

HCPs felt that the Diabetes Questionnaire conveyed that a good life is possible with diabetes and that it supported them to give patients positive feedback related to high scores. However, HCPs also expressed that they found it natural to focus the discussion on low scores, particularly on the dimension related to general well-being. They found it important to discuss these low scores openly, reporting that, sometimes, other topics had to be set aside. Sometimes, low scores were considered by HCPs as being difficult to handle when they were not able to offer help, despite wanting to do so. Although some HCPs doubted their own competence in addressing some of the issues raised through the questionnaire, some also expressed that, sometimes, it was enough just to listen and acknowledge low scores, convey that it is okay to not feel good, and confirm what they can do to support. Low scores could also reveal the need for patients to be referred to a psychologist or welfare officer; however, some HCPs were concerned about being unable to make some of these referrals.

Patients and HCPs valued the way in which dialogue about experiences of support in diabetes care was initiated by the Diabetes Questionnaire. Some patients stated that the questionnaire provided acknowledgment that they had the support they needed from diabetes care. For others, the questionnaire helped to reveal the need for different or increased support. Although some HCPs had expected to learn more, they valued instances in which the potential for improvement was indicated. The questionnaire initiated constructive discussions about the need for medical devices or being referred to a dietician, frequency of clinical visits to the diabetes nurse and physician, distribution of these visits, and continuity in meeting with the same HCPs. Occasionally, the questionnaire led to the patient being invited for clinical visits to another HCP. Among HCPs, it was speculated that it may be difficult for patients to talk about low scores in these dimensions, and some HCPs wondered whether patients dared to be honest. Some patients expressed concerns about how HCPs, as individuals and as diabetes carers, may react to these evaluations and whether they see the results as a basis for constructive improvements or merely as criticism. Some patients suggested that perhaps a different HCP should perform the follow-up if a patient’s scores regarding the experience of support from diabetes care are very low.

### The Process of Initiating the Implementation of the Diabetes Questionnaire

#### Differences in Engagement Among Health Care Management and Coworkers

The extent to which HCPs described the implementation of the Diabetes Questionnaire as a team effort or the work of a few people or an individual varied. HCPs who described implementation as a team effort found it helpful. Discussing and presenting the implementation and results from the Diabetes Questionnaire at team meetings were suggested as necessary strategies for involving the team. However, the predominant experience discussed was being the only person who had to take the lead and do all the work related to the questionnaire by themselves. Some HCPs had chosen to try the Diabetes Questionnaire themselves first to understand whether it would be a strain for others. Others sought to involve their coworkers, but experienced lack of engagement. The lack of engagement was typically described as noninterest or being caused by time constraints rather than them being actively opposed to the Diabetes Questionnaire. Some HCPs who intentionally made a small-scale start found themselves at crossroads, either in terms of involving the rest of the team or discontinuing because it was challenging. Coworkers expressed having much to do and lack of time, which made it difficult to motivate them to add another task:

There are lots of things going on at the clinic: staff are being cut down, there are new routines and everyone feels like there’s no time. So it’s hard to motivate co-workers by saying ‘Spend a little extra time on this.’ You know they’re already struggling with everything else.

HCP, group 3

Patients and HCPs discussed potential differences in interests, roles, and prerequisites between diabetes specialist nurses and physicians in relation to working with the Diabetes Questionnaire. Although a substantial responsibility was taken by enthusiastic diabetes nurses, there were clinics where physicians were actively involved. The central barriers mentioned as being specific to physicians were lack of time because of the large number of patients and the need to attend to many different medical topics during short clinical visits. It was also suggested that there may be lack of interest. Patients reported that the physician’s role regarding the Diabetes Questionnaire could be experienced as unclear. Although some patients proposed that it was reasonable for the diabetes nurse to have the main responsibility, the discussion revealed that it may depend on which physician was involved and that the physician’s participation may be beneficial in the long run.

As experienced by HCPs and suggested by patients, the clinic managers affected the prerequisites for engaging with the Diabetes Questionnaire. Managers with a positive attitude who actively engaged in NDR data and related discussions were valued for their support, whereas managers who did not provide resources or engage were seen as barriers to implementation. Some HCPs doubted that their manager knew about the implementation, whereas others described their manager as being informed but not interested. HCPs discussed time as a resource provided by management. Some HCPs had been able to lengthen their clinical visits, whereas others described their managers as expecting the implementation to be completed with no extra time. Some HCPs had started using the questionnaire and then found themselves lacking the necessary time to prioritize it when there was lot of work to do. The discussion revealed that some managers seemed to consider the Diabetes Questionnaire and related participation in project meetings as being beneficial for the individual HCP rather than being important for the clinic.

#### Starting and Establishing New Routines

Although some participants found it easy, the Diabetes Questionnaire had not yet become part of the established routines for many patients and HCPs. All related activities were new for patients and HCPs (the invitation to complete the questionnaire, completion of the questionnaire, related dialogue, and documentation of questionnaire scores in the patients’ records), highlighting the need to establish new routines. The logistics either for postal invitations or for patients to complete the questionnaire in the waiting room were important steps that needed new routines. Patients and HCPs described that both invitations and completion were easily forgotten and that not all patients realized that they were supposed to complete the questionnaire before the clinical visit. HCPs referred to a need for routines to be locally developed, and some also described difficulties in taking the first step and knowing how to start. Team discussions were emphasized as important for finding and adhering to new routines. HCPs also stressed a need to acknowledge that finding, learning, and incorporating new routines into practice requires time and effort, with some suggesting that it could take several years before using the Diabetes Questionnaire becomes the default approach.

Working with the Diabetes Questionnaire during clinical visits was not seen by HCPs as technically difficult, but rather as a question of people being familiarized with strategies to engage in the necessary dialogue. Although some patients experienced differences in the way in which their clinical visits were organized, these differences were not major. Patients’ experiences varied from adequate, open, and useful dialogue to finding that their responses were given little or no attention. Patients suggested that differences in dialogue may be related to the quality of the relationship between the HCP and the patient. Having confidence in the diabetes nurse or physician was described as making the dialogue easier, whereas having a poor relationship with the HCP or having a novice HCP were seen as barriers:

It might be related to the kind of relationship a patient has with their physician and nurse...If a patient has a bad relationship, then it might be difficult to use the questionnaire.

patient, group 1

HCPs described the different approaches that were applied and found it helpful to discuss how to practically handle the questionnaire and the scores during clinical visits with their peers. Although some HCPs found the questionnaire to be a useful starting point for opening the dialogue, others combined aspects of the questionnaire together with other topics such as medical parameters and educational elements about diet or physical activity. Other HCPs saved the questionnaire for the end of the visit. HCPs who used the questionnaire as a starting point found that this meant that the meeting was directly targeted at the patient’s problems, thoughts, and queries and found this to be more fruitful than conducting it as the last component of the visit. These HCPs let the dialogue be directed by the patient’s scores and what the patient found as most important to talk about at the time. Some HCPs discussed sometimes having missed the completed questionnaires or forgetting to talk about the scores during clinical visits, and some of them felt bad about neglecting the patient’s responses. Other HCPs described forgetting other things in favor of the Diabetes Questionnaire. HCPs who had not invited all patients to participate in the questionnaire reported the need for strategies to remember which patients to ask for responses.

Some HCPs found that the questionnaire saved time during clinical visits, whereas others found that it took more time to do something extra and that it competed with other important aspects of their work. Patients expressed that the discussion related to the questionnaire did not necessarily take a long time. HCPs mentioned that, sometimes, it felt overwhelming to make the time to talk about everything during a clinical visit, suggesting that they were only able to focus on a few topics at each visit. In addition, HCPs reported that, sometimes, it was a difficult balancing act between what the patients wanted to talk about and what information diabetes care is obliged to offer. Some of the HCPs who described the questionnaire as not adding more work still struggled to deal with several different topics during a clinical visit. Some HCPs suggested that other aspects had to be excluded in favor of the questionnaire:

I can’t see anything negative related to the questionnaire. However, because it’s an extra task, there might still be a need to remove something else to make time for it.

HCP, group 2

HCPs hoped for high response rates over time and discussed strategies to encourage patients to understand that the Diabetes Questionnaire was a way to prioritize their perspectives in the operations of the clinic. HCPs suggested that it would be useful to provide more information and reminders for patients, provide reminders to the whole team to talk about the questionnaire with patients, feature the Diabetes Questionnaire in the waiting room, and be in contact with those in transition from pediatric care to diabetes care for adults. Patients reported that insufficient dialogue regarding questionnaire scores during clinical visits gave the impression that there was no point in them completing it. The reasons for this included HCPs forgetting to address the questionnaire, not having looked at the results beforehand, or leaving it as the last thing to be addressed during the visit. Patients mentioned that, sometimes, it was difficult for them to take the lead in ensuring that the questionnaire was discussed.

#### HCPs’ Experiences of Support During Implementation of the Questionnaire

Although HCPs described having access to support from facilitators during the implementation of the questionnaire, not all of them used it. The videos, information, and recommended strategies presented during project meetings were described as instructive, and some HCPs felt that more support was not needed. Those in need of more support found that help from facilitators was easily available via the internet and that the support met their needs. Some HCPs consulted the local information technology department to receive the support they needed to solve practical issues. Among HCPs experiencing lack of support from the managers at their clinic, there was a desire for additional information from the NDR, particularly, information directed to managers to encourage them to sanction this work.

Project meetings, during which HCPs from different clinics came together, were strongly appreciated as being motivational and providing opportunities to discuss and receive advice from peers regarding administrative and practical solutions. HCPs expressed a desire for more peer support, which was suggested as a potential means of supporting the dissemination of the questionnaire to coworkers at the clinic. Organizing peer meetings was not expected from the project facilitator, but was considered as something that the HCPs could, and did, arrange by themselves:

We’re going to have a collaborative meeting to compare notes and learn about what the others have done. We’re going to get some ideas about how to move forward with a few things.

HCP, group 1

#### Pros and Cons Regarding the Questionnaire and Its Items and Dimensions

Patients’ general perceptions varied from seeing the Diabetes Questionnaire as a useful tool for highlighting their perspectives to a general reluctance toward questionnaires and their results. This variation corresponded to HCPs’ perceptions of their patients’ views. Some patients described the questionnaire as a tool for reflecting on their own situation in a new way. In positive terms, patients expressed that the results could strengthen their self-esteem and the feeling that they were handling their situation well. However, concerns were raised about the opposite outcome if the questionnaire emphasized their difficulties:

When I looked at the scores, I felt like I was doing very well. This can fortify your self-esteem, and make you feel like things aren’t so bad after all. But it can also be the other way around.

patient, group 4

Patients expressed that they appreciated the digital format, which enabled the results to be directly viewed and automatically transferred to the system. In addition, they felt that, sometimes, a printed copy may be useful for remembering what was said. In general, patients found the items relevant and easy to respond to, even though the relevance of some items and the total number of items could be questioned from an individual perspective. Some patients felt that there were too few response alternatives for some items and that it was difficult to choose between them. Patients also mentioned the difficulty of grading a feeling and concerns about the undue influence of factors that were unrelated to diabetes or their current state on the day when answering the items.

HCPs felt familiar with dialogue at the dimension level. Patients and HCPs found that dimension scores made it easier to identify areas in which there was scope for improvement. The dimensions were generally found to confirm the patient’s experience; however, scoring was sometimes questioned by patients for not matching their responses and giving an overly negative picture. HCPs sometimes found that their patients paid much attention to the actual scores, thus inhibiting dialogue related to the contents of the dimensions. HCPs compared the dimension scores with each other, focusing the dialogue on dimensions with low scores. However, some HCPs expressed that it could be difficult to interpret the score levels and determine the level that constituted a low score. Scores that were neither high nor low were considered the most difficult to handle because of concerns about neglecting something important. HCPs experienced situations in which patients interpreted items differently, emphasizing the need for dialogue and individualized approach. HCPs suggested that it would be helpful for the system to show responses from individual items.

#### Administration and Completion of the Diabetes Questionnaire

Although patients generally found the digital format easy to handle without assistance, some asked next of kin for practical assistance. Some patients speculated that older people may have difficulty and suggested that diabetes nurses could provide initial assistance if a patient lacked self-confidence. HCPs believed that there were no technical impediments for their patients to complete the questionnaire. HCPs found the digital format as advantageous and reported that their older patients found it as fun and had higher response rate than younger patients. Most clinics invited their patients to complete the questionnaire before visiting the clinic, whereas some asked their patients to complete the questionnaire in the waiting room. To give time to reflect and provide honest responses, patients expressed a preference for completing the questionnaire at home by themselves in peace and quiet. Patients suggested that it would be useful to have the ability to highlight items that are in need of dialogue upon completion.

The clinics had different approaches regarding which patients were invited to complete the Diabetes Questionnaire. Some clinics invited all patients who were asked to attend a clinical visit. Others described that although the long-term goal was to invite all patients at least once, they aimed for a small-scale start and described different methods of selection. For example, they may select from patients with physician appointments, those invited to the first and last appointments during the day, or those assumed to have the most need. Reasons for nonselection included patients with dementia, those assumed to have difficulties with the digital format, or those known to not speak Swedish. Some HCPs found it difficult to know how to choose patients to invite.

Some HCPs were concerned about what they deemed to be a low response rate and inability to reach those for whom the questionnaire could be most useful. Interested in the reasons for low response rates, the HCPs pondered whether this was related to lack of time or interest or technical difficulty or if the aim of the Diabetes Questionnaire was not clear enough. Suggested strategies for increasing the response rates included explaining the intention of the questionnaire as a clinical tool, offering technical solutions to complete the questionnaire in the waiting room, and the possibility of offering a pen-and-paper version. The possibility of enabling the questionnaire to be completed by patients with visual impairment or those who did not speak Swedish was also suggested.

HCPs reported that the digital NDR tool for administering the Diabetes Questionnaire was easy to use. However, there were local administrative barriers that were time-consuming in some cases when inviting the patients to complete the questionnaire when they were summoned to clinical visits. Although HCPs sought to temporarily solve the administrative routines during this project, they stated a need to overcome these local barriers to enable them to implement the questionnaire as an established routine offered to more of their patients:

I think one of the most important things is how to organize the process to make sure that it works. It’s a practical question of how to send these [questionnaire invitations] to the patients so that patients respond to them. It needs to be simple.

HCP, group 2

#### Future Opportunities and Concerns

Patients and HCPs saw potential positive long-term impacts of using the Diabetes Questionnaire related to patients’ individual needs, HCPs’ professional needs, and group-level assessment of diabetes care. However, participants stressed that some effort from diabetes care was required. Patients emphasized that if they were to consider completing the questionnaire, there must be scope for dialogue about their scores during clinical visits. Similarly, HCPs stressed the importance of being attentive to patients’ scores. Patients and HCPs emphasized the necessity for diabetes care to have the organizational readiness and resources to undertake the actions needed regarding questionnaire outcomes. In addition, patients and HCPs suggested that HCPs may need support for learning how to handle, interpret, and act on questionnaire scores. A patient expressed the following:

What actions are we going to link to these things? How much time do we have? We need to have strategies that are ready to use. There needs to be support for the people who are actually going to handle this.

patient, group 1

Patients were interested in opportunities for individual longitudinal follow-up, possibly related to the changes made. However, they reflected on the extent to which the HCPs had the time required for engaging in dialogue related to the questionnaire on a routine basis, which added to their administrative burden. HCPs who intended to continue using the Diabetes Questionnaire suggested that ways of working may need to be changed to create the time needed. Some HCPs experienced the implementation of the questionnaire as being helped by workplaces making efforts to implement more person-centered care. In addition, some HCPs suggested that implementing the questionnaire added another dimension to the pleasure they experienced in their work, leading to professional development and increased commitment and enjoyment.

Patients and HCPs speculated about the opportunities for and value of cross-sectional and longitudinal group-level analyses following the broad implementation of the questionnaire. HCPs described the potential for actively conducting analysis in the same manner as for the traditional NDR data, with the Diabetes Questionnaire adding new aspects. For quality improvement, HCPs stressed the value of assessment of local data and comparisons with other clinics. Both patients and HCPs stressed that by including the questionnaire as part of the NDR, there was the potential to influence managers and politicians. However, some patients also expressed that the greatest benefits of the questionnaire were related to the dialogue about their individual situation, and they spoke against a strict focus on scores and statistics. Patients suggested the possibility of using the questionnaire to identify patients in need of support with educational activities or sharing experiences with peers. Moreover, some patients raised concerns about the potential for diminished access to care for patients with high questionnaire scores if diabetes care prioritized patients with low scores.

## Discussion

### Principal Findings

The findings of the focus groups in this qualitative study revealed 2 main categories that integrated patients’ and HCPs’ experiences and together formed the overarching theme, *While implementation demands new approaches, the Diabetes Questionnaire provides a broader perspective.* The first main category (*The Diabetes Questionnaire supports person-centered clinical visits*) was based on comments expressing that the digital Diabetes Questionnaire encouraged reflection in preparation for clinical visits, brought important topics to light during clinical visits, and broadened the scope of discussion by providing additional information. The second main category (*The process of initiating the implementation of the Diabetes Questionnaire*) comprised comments that expressed differences in engagement among HCPs and their managers, the challenges associated with establishing new routines, experiences of support during the implementation of the Diabetes Questionnaire, thoughts about the questionnaire, the need to implement local administrative routines, and opportunities and concerns regarding continued use.

### Comparison With Previous Work

#### Overview

During the implementation of PROMs, it is important to consider the needs and perspectives of patients and HCPs [27]. This is the first study focusing on patients’ and HCPs’ experiences of using the digital Diabetes Questionnaire in routine diabetes care clinical visits. In addition to valuable input to the specific project related to Swedish diabetes care and NDR, this study contributes to the collective learning process on the use and implementation of PROMs and PREMs in routine care.

#### Using PROMs and PREMs as Clinical Tools to Support Person-Centered Care

In accordance with previous proposals regarding the clinical use of PROMs [20-24], the current results suggest that the use of the digital Diabetes Questionnaire can support person-centered clinical visits for adults living with diabetes. This confirms the suggested potential benefits from the initial component of this study [29]. Although person-centered care can be defined in different ways, common characteristics involve active patient engagement; partnership; shared decision-making; and the need for care to be respectful of and responsive to individual patient preferences, needs, and values [32,33]. For diabetes care, it has been emphasized that HCPs and patients have a shared responsibility to make person-centered clinical visits possible. A central prerequisite is that both parties be adequately prepared. Patients have an important responsibility to raise topics that are important to them, and HCPs are expected to be up-to-date with each patient’s records and ongoing progress [34]. In this study, the digital Diabetes Questionnaire was found to support reflection and active preparation for patients and HCPs. During clinical visits, the questionnaire helped to bring important and sometimes newly revealed topics to light and strengthened collaboration and mutual participation. Comparable findings were reported in Swedish rheumatology care [35], where the use of PROMs has been found to strengthen patients’ involvement and support interaction and shared decisions between HCPs and patients.

Although strengthening patient perspectives in diabetes care has been a topic of research interest for many years [6-8,16,36], research continues to show a gap between recommendations and patient experiences. Adults with diabetes still describe a lack of person-centered care and a desire for HCPs to understand more about their situation and needs and which actions and approaches of HCPs are most helpful [37]. More structured strategies for incorporating patients’ perspectives and encouraging active patient participation in clinical visits are warranted [38]. As a clinical tool, the Diabetes Questionnaire can provide a helpful step in the direction of systematically strengthening patient perspectives. However, this does not exclude the need for other actions. Initiatives such as digital web-based tools for self-monitoring and interacting with diabetes specialist nurses for self-management support [39] may be well suited for use in combination with the questionnaire.

A unique feature of the Diabetes Questionnaire is that, in addition to elucidating experiences in daily life, it includes experiences of support from diabetes care. In the first part of this study [29], concerns were raised regarding whether patients would be comfortable about being honest and whether HCPs and patients would be comfortable discussing the relevant issues [29]. However, in accordance with previous studies describing the basis for and development of the Diabetes Questionnaire [3,5], the results from this study confirm the value of discussing the extent to which patients experience adequate support from diabetes care. Aspiring for collaboration and partnership, it should be possible to discuss questions such as the extent to which the individual patient experiences the support they need and whether the patient feels able to talk about the topics that are most important to them during clinical visits. However, in cases where PREM scores were very low, patients suggested that it may be appropriate to involve a different HCP in the follow-up.

In Norway, a related project investigated the assessment of diabetes distress in diabetes care for young adults with type 1 diabetes. This previous study used the Problem Areas In Diabetes (PAID) scale in conjunction with an empowerment-based communication manual to guide nurses in reviewing and discussing PAID scores [40-42]. In accordance with the current results, the researchers reported that their approach promoted reflective thinking and dialogue and facilitated patient-provider relationships and person-centeredness [42]. Another similarity between the results of the 2 studies is that the questionnaire scoring was enlightening for HCPs [42,43]. Satisfactory glycemic control can obstruct HCPs’ understanding of the patient’s situation, thus concealing significant challenges they face in everyday life. Questionnaire data can reveal important information about adults with diabetes, for whom the everyday personal cost of well-controlled glucose levels can be high. Similar to PAID scale [42], the Diabetes Questionnaire can be helpful for focusing on individual patient experiences and topics other than medical matters that potentially affect medical outcomes. Another similarity with the Norwegian results [42] is the importance of discussing the patients’ responses and the need for patients to be able to clarify the nuances and rationale behind their responses. Furthermore, excessive focus is sometimes placed on numerical scores. Instead, it may be preferable for questionnaires to be used as conversation starters that make the dialogue more constructive and facilitate participation.

As in the current results, the young adults in the Norwegian project appreciated the enhanced emphasis on their situation and expressed that it was worth the time required to complete the questionnaire as preparation for clinical visits. However, the findings also revealed that completing PAID scale and discussing their responses made patients feel exposed, uncomfortable, and vulnerable and that some items were painful to answer [42]. We did not find similar reactions to the Diabetes Questionnaire in this study. As highlighted in an increasing number of studies [44-48], the careful and reflective use of language is important in diabetes care, and the words used can impact how individuals view diabetes and themselves. During the development of the Diabetes Questionnaire, special effort was made to reflect the phrasing used by adults living with diabetes and to avoid being disrespectful or offensive or adding to the burden of diabetes [2,3]. In this study, the Diabetes Questionnaire was found to encourage the idea that a good life is possible with diabetes and support HCPs in giving positive feedback to patients. However, during clinical visits, HCPs found it natural to focus on dimensions with low scores and felt that it was important to do so openly. In addition to the positive statements from patients, some participants remarked that there may also be a risk of emphasizing the difficulties. This risk will be important to be examined in more detail in future studies. In addition to differences between the 2 questionnaires’ content or wording, differences in experiences may also be related to practices regarding discussion of patient experiences in clinical visits or the specific focus on young adults with type 1 diabetes in the Norwegian studies [40-42]. Another related initiative is the recently announced Danish implementation of a nation-specific digital tool for patient-reported outcomes [49]. Similar to this study, the researchers targeted adults with type 1 and type 2 diabetes more broadly.

#### Implementing PROMs and PREMs in Routine Practice

The current results have many similarities to the facilitators and barriers to implementing PROMs and PREMs in organizations delivering health-related services identified in a review of reviews reported by Foster et al [27] and in a summary of case studies reported by Stover et al [50]. In accordance with the current results, a central message is that integration into routine care requires effort and time [27,50]. Central traits that have been reported to facilitate implementation include the experience of specific PROM or PREM measures as a meaningful and useful approach for strengthening patient perspectives. Another important trait is the existence of evidence that these tools have satisfactory measurement quality [27,50]. Consistent with the findings of previous studies [1-4,29], the current results add to the increasing evidence suggesting that the Diabetes Questionnaire possesses the necessary central traits.

In addition to these central traits, the identified facilitating characteristics for PROMs and PREMs include application at the individual level, absence of license costs, user-friendly technical systems, and directly and easily available data. Further facilitating characteristics include the possibility to adapt data collection and clinical use to organizational work processes and appointment schedules [27,50]. The Diabetes Questionnaire is intended for use at the individual level, and there is no license cost for clinics connected to the NDR. This study shows that the provided digital tool was easy to use for data collection and presentation of scores. However, HCPs experienced barriers related to the local administrative procedures and systems for invitation. Similar to the findings reported by Stover et al [50], the HCPs in our study suggested that these administrative barriers needed to be resolved locally to fit each clinic’s resources, existing routines, technical systems, and workflows.

Currently, there is lack of information regarding the potential need to prepare patients for the use of PROMs [27]. Patients in our study found the digital questionnaire easy to use, and special training other than information from their HCP was not requested. However, it was suggested that the diabetes nurse could potentially be of assistance for the first time the questionnaire is introduced. The participating patients in this study received information about the intentions of using the Diabetes Questionnaire and the data collection process during an introductory meeting for the study. Consequently, future evaluations are needed to determine whether the information provided by HCPs during clinical visits is sufficient. A potential negative aspect related to digital PROMs highlighted in a review by Meirte et al [24] is that some patients, particularly those who are older, may have difficulties in using technology. This was also suggested in our focus groups with patients; however, it was not directly experienced by our participants. In contrast, HCPs in our study reported that their older patients had higher response rate than their younger patients. Similar to Meirte et al [24], our focus groups suggested that a paper version could be offered to those who were less familiar with technical tools.

Some HCPs found it natural to integrate the Diabetes Questionnaire into the dialogue, reporting that it did not necessarily take more time and, possibly, even saved time. However, as described by Stover et al [50], we found that it could be challenging for HCPs to know how to initiate related dialogue. This dialogue was also experienced as interfering with other responsibilities during the limited time available during clinical visits. Barriers related to competing priorities and worries regarding workload have also been described in previous studies [43,50]. In the Norwegian project using PAID scale in diabetes care mentioned previously [43], substantial challenges were described regarding time and resources and the need to balance between addressing patients’ emotional concerns and HCPs’ other duties. The competing responsibilities described were mainly technical issues for diabetes nurses and biomedical issues for physicians [43]. While patients in our study clearly stressed the need to discuss their questionnaire scores, concerns were raised about whether the HCPs would have the time needed on a routine basis. Patients expressed that the main benefits of the questionnaire were related to the clinical dialogue about their individual situation. Similar to the previously reported barriers regarding group-level monitoring of PROM data alone [27], patients in our study questioned the benefits of completing the questionnaire if there was no related dialogue. Potential benefits of cross-sectional and longitudinal group-level analyses and quality improvement informed by PROM and PREM data were discussed by both patients and HCPs. However, the patients expressed that regardless of the value of the data, this should only be seen as an additional benefit of broad implementation at the individual level, rather than being the main objective.

The implementation of the Diabetes Questionnaire was predominantly taken on by small groups or solitary enthusiastic individuals. The engagement and support experienced from coworkers and managers varied. This does not appear to be a unique situation. According to Foster et al [27], the main workload often falls on a few members of the working team. The current results also revealed that HCPs who described team effort and engaged support from their manager found this situation helpful. Contextual factors such as leadership, organizational culture, and readiness for change have been reported in several implementation frameworks to influence implementation [51]. In situations where the implementation process is proposed by the organization, it has been recommended that the manager needs to be engaged to motivate the use of PROMs and lead the implementation process [27]. However, there is a knowledge gap regarding cases in which clinicians want PROMs to be implemented but the organizational culture or manager is not receptive to change [27]. In this study, the managers had to agree to their clinic’s participation. However, the wish to implement the Diabetes Questionnaire generally came from HCPs and not from their managers. Some HCPs described their managers as being genuinely engaged. However, some HCPs described managers who did not consider the implementation to be sufficiently important for the clinic to invest time in, but rather as being beneficial for the individual HCP, who should be thankful for being allowed to implement it. Integrating patients’ perspectives in clinical visits and outcome assessments of care at the individual and group levels is recommended in the guidelines for diabetes care [6-15]. The current results support previous reports [27,43] that the use of PROMs often comes with conditions, requiring the capacity and resources to handle the responses in individual clinical visits and health care organizations and in the long term [27,43]. The implementation of the questionnaire cannot rely on solitary enthusiastic individuals and should not be seen as a measure that only benefits HCPs. Clinic managers, decision makers, and health care organizations need to provide prerequisites and support for HCPs to be able to focus on the emotional aspects of diabetes. To achieve this goal in routine care, considerable amount of important work remains to be done.

### Methodological Considerations

To strengthen the credibility of the current findings, we included participants with various perspectives [52-54]: patients, specialist nurses, and physicians working with diabetes at different hospital-based clinics or in primary care. The focus groups [55] generated nuanced and rich data from discussions that led participants to reflect on their different or shared experiences and thoughts. A limitation of this study was that patients had less experience in using the Diabetes Questionnaire than HCPs, and our results may more strongly reflect the perspectives of individuals who felt more positively about the Diabetes Questionnaire. To strengthen credibility and address dependability, researcher triangulation [52-54] was conducted throughout the analysis, thoroughly discussing each step to gain a shared understanding and avoid misinterpretation of the data. Together, the research group (all were women) has considerable collective experience in qualitative research and diabetes care, including the perspectives of both registered nurses (EL, JL, MSE, and UBJ) and a physician (KEO). EL (registered nurse) and KEO (PhD) work at the NDR; EL as a development manager and KEO as the director. KEO also works as a consultant in diabetes care and with clinical research. JL (associate professor), MSE (PhD), and UBJ (professor) teach in higher education and conduct clinical research at universities. All members of the research group have been involved in the previous development process of the Diabetes Questionnaire in various ways. The research group had no established relationship with the participants before the study. For the reader to be able to judge the transferability to other settings, we strived for transparency and rich descriptions of results.

### Implications and Future Perspectives

The long-term goal is for the digital Diabetes Questionnaire to be used as a clinical tool to strengthen patient perspectives in routine diabetes care and to be considered together with medical variables in the Swedish NDR. For this goal to be realized, there is considerable amount of work to be done. Use at the individual level is the foundation of implementation. On the basis of the current results and advice from researchers such as Foster et al [27], ongoing and future studies will be required to evaluate whether a further developed implementation strategy including clear advice for inviting all patients at the clinics; more formally appointed implementation leaders; and more formal, structured, and recurring involvement of clinicians, coworkers, and clinic managers could result in greater collective effort and a clear mandate for change. This study focused on the initial experiences of initiating the use of the Diabetes Questionnaire. It is also important to study the long-term impact of the questionnaire by focusing on experiences from recurrent use, particularly from patients’ perspectives. In addition, it is important to consider a long-term perspective on the implementation process. Guided by normalization process theory [56-59], in future studies, we plan to focus on the support and strategies needed to embed the use of the Diabetes Questionnaire as a natural and continuous part of routine clinical diabetes care. Long-term use presents opportunities for longitudinal follow-up at the individual level and sufficient data for group-level analysis as the basis for quality improvement. Being part of the NDR, this will also enable evaluations combining PROM and PREM data with medical variables. These opportunities and potential benefits from continued use of the Diabetes Questionnaire were expressed by patients and HCPs. We aim to evaluate these possibilities in future studies. These potential outcomes are also consistent with increasing call for patients’ perspectives to play a greater role in assessing outcomes of diabetes care and to be incorporated into diabetes registries [60,61].

The NDR has comprehensive long-term experience in secure data management of medical variables. Since the start of the PROM and PREM project, the NDR has continuously sought to ensure that technical solutions conform to regulations and that patients’ questionnaire data are handled in a secure manner. As addressed by Meirte et al [24], these aspects are essential for making broad and long-term implementations in routine care possible. Together with practical issues related to the different digital systems used in health care organizations, the security, lawfulness, and feasibility of data handling continue to be highly important factors.

### Conclusions

The Diabetes Questionnaire can broaden the scope of health data in routine diabetes care. While implementation demands new approaches, patients and HCPs saw potential positive impacts of using the questionnaire at both the individual and group levels. These results can inform further development of implementation strategies to support the clinical use of the questionnaire.

 

## Appendix

Multimedia Appendix 1 Interview guides used for focus group discussions with health care professionals and patients.

## Abbreviations

HCP: health care professional
NDR: National Diabetes Register
PAID: Problem Areas In Diabetes
PREM: patient-reported experience measure
PROM: patient-reported outcome measure
