# Supplementary material for: Implementing the Digital Diabetes Questionnaire as a Clinical Tool in Routine Diabetes Care: Focus Group Discussions With Patients and Health Care Professionals
Source: JMIR Diabetes. 2022 May 25;7(2):e34561. doi: 10.2196/34561 (PMC9178456; doi:10.2196/34561)
Supplement: Multimedia Appendix 1 [file diabetes_v7i2e34561_app1.pdf]

Supplementary material to
Svedbo Engström et al. Implementing the Digital Diabetes Questionnaire as a Clinical Tool in Routine Diabetes Care: Focus Group Discussions with Patients and Health Care Professionals

Multimedia Appendix 1. Interview guides used for focus group discussions with health care professionals and patients

(Translated into English for publication reasons only. The focus group discussions were held in Swedish.)

Interview guide for focus group discussions with health care professionals

Experiences related to the Swedish National Diabetes Register (NDR) and experiences from initiating the use of the digital Diabetes Questionnaire

How was your experience of starting to use the digital questionnaire?

Can you tell us about how the use of the digital questionnaire was initiated at your clinic? How did you plan the implementation? How are the tasks distributed among you and your co-workers? Can you give some examples?

What role did the clinic manager play in initiating the use of the questionnaire at your clinic?

What role did your co-workers play in initiating the use of the questionnaire at your clinic?

What support did you receive when initiating the use of the questionnaire?

Did you experience a lack of support?

Did you experience any barriers when initiating the use of the questionnaire?

Did you lack any resources when initiating the use of the digital questionnaire?

From a strictly practical point of view, how has your experience of using the digital questionnaire been?

In what ways have you used the patient-reported information obtained with the questionnaire (e.g., information about how the patient is feeling, how they manage their diabetes, and their experiences of support from diabetes care)?

Did you feel sufficiently competent to use the digital tool for administering the questionnaire, inviting the patient to complete the questionnaire, receiving the patient’s answers, and using the questionnaire during the clinical visit?

How have you handled situations in which patients have low scores on the questionnaire dimensions (including the PROM and PREM dimensions)?

Has the questionnaire led to any changes (positive or negative) in diabetes care?

Points of view and attitudes regarding the questionnaire and the use of patient-reported information after initiating the use of the digital Diabetes Questionnaire

What are your positive and negative experiences of using the digital questionnaire?

In what ways do you think clinical visits have been affected by using the questionnaire?

What do you think are the advantages and disadvantages of using the Diabetes Questionnaire regarding the questionnaire itself, and regarding the information obtained using the

questionnaire (how the patient is feeling, how the patient manages their diabetes, and the patient's experiences of support from diabetes care)?

In what ways do you think diabetes care can be developed using the responses from the Diabetes Questionnaire?

In what ways do you think that you can undertake professional development using the responses from the questionnaire?

What are your thoughts in relation to the concept of participation in diabetes care?

Have your thoughts in relation to the concept of participation in diabetes care changed as a result of implementing the questionnaire? Can you give some concrete examples of participation related to the Diabetes Questionnaire?

How have the patients' participation and control over their health and diabetes care been affected by the questionnaire?

Concluding remarks

Is there anything else you would like to add?

What was the most important thing we talked about today?

Interview guide for focus group discussions with patients

Experiences related to the NDR and experiences from initiating the use of the digital Diabetes Questionnaire

What do you know about the NDR? How do you use the NDR?

In your opinion, what are the advantages and disadvantages of the NDR?

What are your thoughts about using digital technology in diabetes care?

Experiences of using the digital Diabetes Questionnaire

What are your experiences of using the digital questionnaire? Are your experiences positive or negative? In what way? Please specify a concrete example.

Can you tell us how you were shown your questionnaire responses?

Did you experience any problems when answering the questionnaire?

From a strictly practical point of view, how has your experience of using the digital questionnaire been?

How have you used the information from the questionnaire together with your doctor or diabetes nurse?

Points of view and attitudes regarding the questionnaire and the use of the patient-reported information after initiating the use of the digital Diabetes Questionnaire

In what ways do you think the clinical visit is affected by using the questionnaire?

What do you think are the advantages and disadvantages of using the questionnaire?

In what way do you think diabetes care can be developed using the responses from the questionnaire?

Have your thoughts in relation to the concept of participation in diabetes care changed as a result of using the questionnaire? Can you give some concrete examples of participation related to the questionnaire?

How can your participation and control over your health and diabetes care be affected by the questionnaire?

In your view, has the structuring of the clinical visit been changed by using the questionnaire?

In what ways do you think you can undertake personal development using responses to the questionnaire?

Concluding remarks

Is there anything else you would like to add?

What was the most important thing we talked about today?
